# 294. Surveillance for Potential Post-Acute COVID-19 Syndrome Medical Complications in the Emergency Department (ED) – A Retrospective Longitudinal Study of ED Patients Who Had Evidence of SARS-CoV-2 Infection Versus Those Who Did Not

**DOI:** 10.1093/ofid/ofab466.496

**Published:** 2021-12-04

**Authors:** Isabel Lake, Richard C Wang, Richard E Rothman, Oliver Laeyendecker, Reinaldo Fernandez, Gaby Dashler, Thomas Quinn, Yu-Hsiang Hsieh

**Affiliations:** 1 Johns Hopkins School of Medicine, Baltimore, Maryland; 2 Johns Hopkins University School of Medicine, Baltimore, MD; 3 Johns Hopkins University, Baltimore, Maryland; 4 NIAID, Bethesda, Maryland

## Abstract

**Background:**

As the COVID-19 pandemic continues, growing attention has been placed on whether patients previously infected with SARS-CoV-2 have an increased risk of developing and/or exacerbating medical complications. Our study aimed to determine whether individuals with previous evidence of SARS-CoV-2 infection prior to their current emergency department (ED) visit were more likely to present with specific clinical sign/symptoms, laboratory markers, and/or clinical complications.

**Methods:**

A COVID-19 seroprevalence study was conducted at Johns Hopkins Hospital ED (JHH ED) from March 16 to May 31, 2020. Evidence of ever having SARS-CoV-2 infection (PCR positive or IgG Ab positive) was found in 268 ED patients at this time (i.e. infected and/or previously infected). These patients were matched 1:2 to controls, by date, to other patients who attended the JHHED. Clinical signs/symptoms, laboratory markers, and/or clinical complications associated with ED visits and/or hospitalizations at JHH within 6 months after their initial ED visit was abstracted through chart review for these 804 patients. Cox proportional hazards regression analyses were performed.

**Results:**

Among 804 ED patients analyzed, 50% were female, 56% Black race, and 15% Hispanic with a mean age of 47 years. 323 (40%) patients had at least 1 subsequent ED visit and additional 70 (9%) had been admitted to JHH. After controlling for race and ethnicity, patients with evidence of current or prior COVID-19 infection were more likely to require supplemental oxygen [hazards ratio (HR) =2.53; p=0.005] and have a cardiovascular complication [HR =2.13; p=0.008] during the subsequent ED visit than the non-infected patients.

**Conclusion:**

Our findings demonstrate that those previously infected with SARS-CoV-2 have an increased frequency of cardiovascular complications and need for supplemental oxygen in ED visits in the months after their initial SARS-CoV-2 infection was detected. EDs could serve as a critical surveillance site for monitoring post-acute COVID-19 syndrome complications.

**Disclosures:**

**Richard E. Rothman, PhD, MD**, **Chem bio** (Grant/Research Support)

